# Mitochondrial Dysfunction and Chronic Liver Disease

**DOI:** 10.3390/cimb44070218

**Published:** 2022-07-09

**Authors:** Chunyan Zhang, Yabin Zhao, Mengli Yu, Jianru Qin, Bingyu Ye, Qiwen Wang

**Affiliations:** 1 State Key Laboratory Cell Differentiation and Regulation, College of Life Science, Henan Normal University, Xinxiang 453007, China; zcy1119sdc@163.com (C.Z.); zhaoyabinzyb@163.com (Y.Z.); yu15738237855@163.com (M.Y.); 2019020@htu.edu.cn (J.Q.); 2 Henan International Joint Laboratory of Pulmonary Fibrosis, College of Life Science, Henan Normal University, Xinxiang 453007, China; 3 Henan Center for Outstanding Overseas Scientists of Pulmonary Fibrosis, College of Life Science, Henan Normal University, Xinxiang 453007, China; 4 College of Life Science, Henan Normal University, Xinxiang 453007, China; 5 Institute of Biomedical Science, Henan Normal University, Xinxiang 453007, China; 6 Overseas Expertise Introduction Center for Discipline Innovation of Pulmonary Fibrosis, College of Life Science, Henan Normal University, Xinxiang 453007, China

**Keywords:** mitochondrial dysfunction, drug-induced liver injury, alcoholic fatty liver, non-alcoholic fatty liver, hepatocellular carcinoma, viral hepatitis

## Abstract

Mitochondria are generally considered the powerhouse of the cell, a small subcellular organelle that produces most of the cellular energy in the form of adenosine triphosphate (ATP). In addition, mitochondria are involved in various biological functions, such as biosynthesis, lipid metabolism, oxidative phosphorylation, cell signal transduction, and apoptosis. Mitochondrial dysfunction is manifested in different aspects, like increased mitochondrial reactive oxygen species (ROS), mitochondrial DNA (mtDNA) damage, adenosine triphosphate (ATP) synthesis disorder, abnormal mitophagy, as well as changes in mitochondrial morphology and structure. Mitochondrial dysfunction is related to the occurrence and development of various chronic liver diseases, including hepatocellular carcinoma (HCC), viral hepatitis, drug-induced liver injury (DILI), alcoholic fatty liver (AFL), and non-alcoholic fatty liver (NAFL). In this review, we summarize and discuss the role and mechanisms of mitochondrial dysfunction in chronic liver disease, focusing on and discussing some of the latest studies on mitochondria and chronic liver disease.

## 1. Introduction

Chronic liver disease is caused by viral hepatitis, alcoholic fatty liver disease (AFL), non-alcoholic fatty liver disease (NAFL), drug-induced liver injury (DILI), hepatocellular carcinoma (HCC), and other different etiologies, and the course of disease generally lasts for at least 6 months [1,2]. Some studies have focused on the pathogenesis of chronic liver disease, providing some theoretical support for its prevention and treatment [3]. Mitochondria are abundant in the liver, accounting for 13–20% of the liver volume [4], and are involved in the occurrence and development of many liver diseases [5]. Therefore, it is of great significance to explore the relationship between mitochondrial dysfunction and chronic liver disease for the treatment of chronic liver disease.

## 2. Biological Characteristics of Mitochondria

Mitochondria are semi-autonomous intracellular organelles that are essential for the physiological activities of cells. They are not only the “powerhouse” of the cell but also where many important metabolic processes take place [6]. For example, they are the site of intracellular oxidative phosphorylation and adenosine triphosphate (ATP) synthesis, and they exhibit extensive important biological functions, such as energy conversion, tricarboxylic acid cycle, cellular calcium concentration regulation, lipid metabolism, and cell signal transduction [7,8]. In addition, mitochondria are also the main source of intracellular reactive oxygen species (ROS) and the regulatory center of apoptosis [9]. Therefore, maintaining the normal structure and function of mitochondria is crucial for the normal functioning of cells and human health.

## 3. Mitochondrial Dysfunction

**Figure 1:**
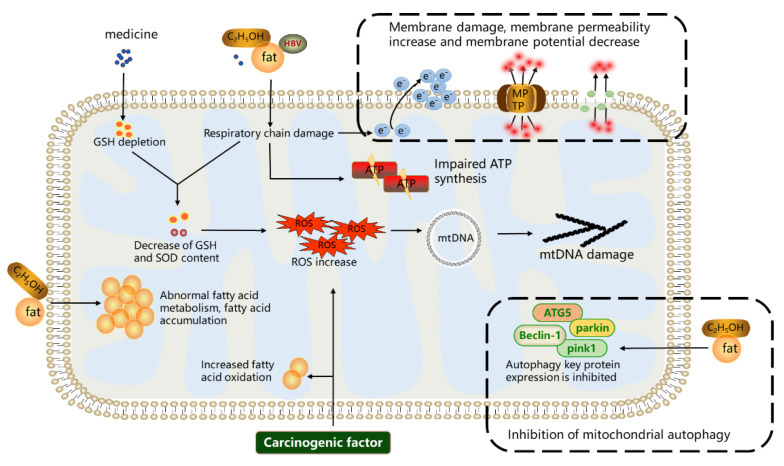
Overview of mitochondrial dysfunction discussed in this paper. During chronic liver disease, various factors can lead to classical changes, including the morphological structure, increased ROS, damaged mtDNA, abnormal energy metabolism, and autophagy within mitochondria.

Mitochondria can be affected by various damage factors, including drugs, viruses, fat accumulation, and carcinogenic factors [3]. When the damage factors exceed the capacity of mitochondria, they can result in abnormal mitochondrial structure and function, which are mainly manifested in the following aspects. (1) Morphological and structural changes: mitochondria swell and lose their original tubular or spherical structure; there is decreased membrane potential and increased membrane permeability; the inner membrane bulge disappears [10]. (2) Abnormal energy metabolism: enzymes and the respiratory chain complexes are inhibited, and ATP synthesis is reduced, leading to an insufficient energy supply [11]. (3) Increased ROS: the ROS scavenging system is disrupted, and as a result, ROS are accumulated in the mitochondria [5,12]. (4) Mitochondrial DNA (mtDNA) damage: mtDNA is exposed without histone protection and is easily damaged by ROS [10,11]. (5) Abnormal mitophagy: the damaged mitochondria cannot be removed in time and are accumulated in cells, causing cell damage and abnormal autophagy [13,14]. In this section, the effects of multiple damaging factors, such as drugs, viruses, fat accumulation, and carcinogens on mitochondria, are summarized in Figure 1.

## 4. Mitochondrial Dysfunction and Chronic Liver Disease

The existing studies on mitochondria mainly focus on exploring the link between mitochondrial dysfunction and disease, observing mitochondrial changes in diseases, and treating diseases by targeting mitochondrial function. Typically, the liver is an important metabolic organ of the body, while mitochondria are the center of material and energy metabolism. Therefore, it is significant to explore the relationship of mitochondrial dysfunction in the liver with the occurrence and development of chronic liver diseases.

### 4.1. Mitochondrial Dysfunction and DILI

DILI is another kind of liver disease caused by various prescriptions or over-the-counter (OTC) Chinese and Western medicines as well as their metabolites in the body [15]. The mechanism of DILI is related to mitochondrial damage in liver cells. Acetaminophen (APAP), diclofenac, aspirin, ibuprofen, and other drugs can cause mitochondrial damage in liver cells [16,17]. Among them, APAP is one of the most common drugs to cause liver injury. It is widely used as a pain reliever but often leads to serious liver damage because of overdose [18].

APAP is mainly metabolized in the body via glucuronidation and sulfation, but a small part (5–10%) of it is converted into the active metabolite N-acetyl-p-benzoquinoneimine (NAPQI) under the action of cytochrome P450 [19]. NAPQI can react with protein sulfhydryl groups to form NAPQI–protein complexes [20]. A normal dose of NAPQI complex can be cleared by glutathione (GSH), but an overdose of APAP will lead to the excessive production of NAPQI complex, which depletes GSH and damages mitochondria [21,22]. Guyen et al., found that the administration of multiple sub-toxic doses (150 mg/kg) of APAP into mice resulted in the accumulation of NAPQI–protein complexes in mitochondria and induced mitochondrial oxidative stress (OS). This process was amplified by the JNK pathway and further led to the opening of mitochondrial permeability transition pores (MPTPs), decreased membrane potential, and inhibited ATP synthesis, finally resulting in mitochondrial dysfunction and liver cell death [23]. In addition, excessive APAP can also lead to the degradation of lysosomes and the release of Fe^2+^, which is subsequently transferred into mitochondria by the mitochondrial calcium ion transporter, eventually changing the permeability of and depolarizing the mitochondrial membrane and causing mitochondrial dysfunction [24]. At present, the treatment methods for APAP-induced liver injury include drugs such as N-acetylcysteine and 4-methylpyrazole [25,26,27], mitochondrial transplantation therapy [28], and other mitochondria-targeting treatments. The characterizations of mitochondrial dysfunction in DILI are summarized in Table 1.

### 4.2. Mitochondrial Dysfunction and Nonalcoholic Fatty Liver Disease (NAFLD)

NAFLD is a clinicopathological syndrome characterized by excessive fat deposition in liver cells, which is caused by factors other than alcohol and the well-established liver-damaging factors [29]. Relevant studies have shown that the main feature of NAFLD is massive fat accumulation in liver cells, resulting in abnormal fatty acid oxidation, a significant increase in mitochondrial ROS, and changes in the mitochondrial membrane lipids and membrane proteins [30,31,32]. Moreover, the impairment of mitochondrial structure and function further exacerbates NAFLD progression.

In the course of NAFLD, fatty acid oxidation is abnormal with the accumulation and saturation of lipids in liver cells. Zeng et al., found that fatty acid translocase (FAT/CD36) on the mitochondrial membrane was heavily palmitoylated in NAFLD, which reduced its ability to transfer long-chain fatty acids into mitochondria and inhibited fatty acid oxidation [33]. In addition, the lipid composition of mitochondrial membranes was altered with the continuous accumulation of lipids. The same result was obtained by Manon et al., who discovered that the contents of cardiolipin, phosphatidylethanolamine, phosphatidylcholine, phosphatidic acid, and other components of mitochondrial membrane lipids in liver cells were changed with the occurrence of NAFLD, causing changes in membrane permeability, membrane lipids, and mitochondrial respiratory chain complexes, as well as subsequent OS and mitochondrial dysfunction [32]. Therefore, the mitochondrial mechanism in the treatment of NAFLD has become a research hotspot. As found by Torres et al., methylation-regulating J protein (MCJ) deletion on the mitochondrial inner membrane reduced lipid accumulation, attenuated steatosis, enhanced the activity of respiratory chain complexes I, and promoted supercomplex formation in the livers of methionine-choline deficient diet-induced NAFLD mice, thereby inhibiting electron leakage and ROS increase [34]. Furthermore, according to Song et al., the activation of AMPK signaling promoted mitochondrial biosynthesis and energy metabolism, reduced ROS levels, maintained mitochondrial homeostasis, and alleviated liver injury in the livers of high-fat diet (HFD)-induced NAFLD mice [35]. These studies have preliminarily demonstrated the feasibility of targeting mitochondria to treat NAFLD. Typically, such feasibility was further verified by Geng et al., who found that carnosol (CAR) was activated by mitochondrial peroxireductase3 (PRDX3), which then inhibited mitochondrial OS, prevented mitochondrial dysfunction and apoptosis, and protected mitochondria in the normal murine hepatocytes of HFD-fed mice and palmitic acid-treated mice, thereby alleviating NAFLD [36]. In addition, Gao et al., reported that baicalin reduced mitochondrial ROS through its own antioxidant capacity, relieved OS, maintained normal mitochondrial morphology and membrane integrity, and protected mitochondrial structure in a tissue engineering model of NAFLD constructed based on HepG2 cells, eventually delaying NAFLD progression [37]. The characterizations of mitochondrial dysfunction in NAFLD are summarized in Table 2.

### 4.3. Mitochondrial Dysfunction and AFLD

Alcoholic fatty liver disease (AFLD) is caused by long-term heavy drinking [38]. In AFLD, liver mitochondria undergo various changes, such as increased ROS levels, decreased mitochondrial membrane potential (MMP), abnormal fatty acid β-oxidation, and abnormal mitophagy [39]. Therefore, investigating the mitochondrial structure and function is crucial for the prevention and treatment of AFLD.

AFLD is mainly characterized by fat accumulation in the liver. Mitochondria serve as the site of fatty acid beta oxidation, and their dysfunction is closely related to fat accumulation. In AFLD patients, together with alcohol-fed mouse models and AML-12 cell models, Torres et al., discovered that alcohol activated casein kinase (CK2), phosphorylated methionine acyltransferase α1 (MATα1), promoted the interaction of MATα1 with peptidyl prolyl cis-trans isomerase 1 (PIN1), and inhibited the MATα1 concentration in mitochondria. As a result, MATα1 was unable to participate in the mitochondrial tricarboxylic acid cycle, oxidative phosphorylation, and fatty acid β-oxidation, finally resulting in fat accumulation and mitochondrial dysfunction [40]. As discovered by Zhao et al., in the livers of alcohol-fed rats, decreased antioxidant enzymes, such as GSH and CAT, increased lipid peroxidation products, and lipid metabolic disorders were observed. Fucoidan pretreatment restored the normal levels of antioxidant enzymes, alleviated oxidative damage, and improved the lipid metabolic disorders [41], indicating that fucoidan prevented the occurrence of FALD to a certain extent. Ma et al., reported the therapeutic effect of resveratrol on rat FALD and found that ROS levels in the livers of FALD rats were controlled after the administration of resveratrol, which in turn improved FALD [42]. In addition, Jiang et al., indicated that mitophagy was also inhibited in the livers of AFLD mice. If the damaged mitochondria were not cleared in time, lipid accumulation also increased in the liver accordingly. In contrast, lipid deposition in the liver was significantly reduced when mitophagy was activated [43]. Zeaxanthin dipalmitate (ZD), a commonly used active antioxidant substance, modulated the autophagy-related AMPK-FoxO3a pathway via P2 × 7 and adiponectin receptor 1 (adipoR1) on the cell membrane and restored the ethanol-inhibited mitochondrial autophagy [44].

In addition, cytochrome P450-2E1 (CYP2E1), an important inducer of intracellular oxidative free radicals, has been found to play an important role in the occurrence and development of AFLD. CYP2E1 is up-regulated in alcohol-treated hepatocytes or animal models. At present, CYP2E1 is recognized as an important cause of ethanol-induced OS and liver injury, and the increased expression of CYP2E1 after alcohol consumption leads to increased ROS production, GSH depletion, decreased MMP, and ultimately, hepatocyte death [45,46]. Li et al., found that the water extract of tea well inhibited the expression of CYP2E1, enhanced the activities of SOD and GSH, increased the content of GSH, and reduced the level of malondialdehyde (MDA) in the livers of AFLD mice, thereby inhibiting AFLD progression [47]. The characterizations of mitochondrial dysfunction in AFLD are summarized in Table 3.

### 4.4. Mitochondrial Dysfunction and HCC

HCC is a common pathological subtype of primary liver cancer, accounting for about 75% of all primary liver cancer cases. HCC has a low survival rate, with a 5-year relative survival rate of only 18% [48]. Therefore, the diagnosis and treatment of HCC have become a research hotspot. According to research data from the PubMed database, a substantially increasing number of studies have been conducted to explore the relationship between mitochondrial dysfunction and HCC in the past few years [49], fully demonstrating that the link between HCC and mitochondrial dysfunction has attracted the attention of many researchers. Mitochondria can participate in the progression of HCC by regulating energy metabolism, redox balance, autophagy, and apoptosis.

Mitochondrial dysfunction mediates the accumulation of ROS and mtDNA damage, which may lead to the development of HCC. As found by Shetty et al., mitochondrial ROS levels doubled in the course of nitrosodiethylamine (NDEA)-induced HCC in mice, leading to DNA damage and proto-oncogene activation, which in turn promoted tumorigenesis [50,51]. In tumor cells, the inhibition of mitochondrial activity can transform these cells into tumor cells with mitochondrial dysfunction (P0), thus resulting in increased stem cell properties and stronger division ability. This phenomenon was demonstrated by Han et al. They inhibited mitochondrial activity in Hep3B liver cancer cells, turning them into Hep3B cells with mitochondrial dysfunction (Hep3B/P0). According to their results, the stem cell properties of Hep3B/P0 cells, such as self-renewal ability, chemotherapy resistance, and angiogenesis ability, were enhanced. These results suggest that mitochondrial dysfunction enabled the transformation of cancer cells into cancer stem cells (CSCs) [52]. In addition, mitochondrial integrity plays an important role in liver cancer cell growth and metabolism. Li et al., found that the over-expression of mitochondrial uncoupling protein inhibited the apoptosis of liver cancer cells by reducing the mitochondrial membrane permeability [53]. Veiga found that phenformin inhibited the mitochondrial respiratory chain complex I (MRCC- I) to inhibit the proliferation of liver cancer cells [54]. Moreover, the inhibition of the mitochondrial ATP synthase e subunit suppressed HCC proliferation [55].

The above studies indicate that mitochondrial dysfunction is involved in the occurrence and development of HCC, and mitochondria might be a therapeutic target for HCC. This was demonstrated by Zhang [56] and Tussy [57] et al., in studies on microRNA (miRNA) targeting mitochondria involved in HCC progression. In the course of treatment for HCC, drugs induced apoptosis and exerted an anti-cancer effect by inhibiting ATP synthesis, promoting increases in the ROS levels, and decreasing the MMP. Hou et al., reported that dehydrocrenatidine (DEC) promoted ROS production, inhibited ATP production, disrupted the MMP, and inhibited the activity of complexes I, III, and IV in the liver cancer cell lines Hep3B and HepG2, as well as in model nude mice. DEC led to mitochondrial dysfunction, promoted cell apoptosis, and exerted anti-tumor effects [58]. Lan et al., discovered that the treatment of HepG2 cells with Aidi injection, an anti-tumor Chinese medicine preparation, regulated the PI3K/Akt and MAPK signaling pathways, collapsed the MMP, and induced the apoptosis of HepG2 cells [59]. In addition, Yao et al., found that oroxylin A (OA) inhibited the activity of cyclin-dependent kinase 9 (CDK9) in HepG2 cells, which in turn inhibited pinK1-PRKN-mediated mitochondrial autophagy, induced the accumulation of damaged mitochondria in HepG2 cells, suppressed the growth of HCC cells, and reduced the resistance of HCC to sorafenib and other anti-cancer drugs [60]. The characterizations of mitochondrial dysfunction in HCC are summarized in Table 4.

### 4.5. Mitochondrial Dysfunction and Viral Hepatitis

Viral hepatitis refers to liver inflammation caused by a viral infection and is a global health problem [61]. Among the five hepatitis viruses, hepatitis B virus (HBV) and hepatitis C virus (HCV) can cause chronic diseases and toxic hepatitis [62]. Notably, HBV infection, a class B infectious disease, is the most prevalent, and it is estimated that 290 million people worldwide suffer from chronic hepatitis B (CHB) [63].

HBV infection is characterized by mitochondrial abnormalities. In hepatitis B patients, mitochondrial morphological and structural changes can usually be detected, including the loss of regular tubular and spherical structures, the disappearance of mitochondrial cristae, and mitochondrial swelling [64]. These mitochondrial abnormalities are closely related to the HBx protein. According to relevant studies, the X protein of HBV is the main viral regulatory protein with diverse activities. The HBx protein can target and bind to the mitochondrial outer membrane, resulting in increased ROS levels and mtDNA damage [65]. Furthermore, HBx protein can also interact with cytochrome C oxidase III (COXIII), impairing ATP synthesis [66]. This was demonstrated by Ling et al., who constructed an HBx-expressing mouse model and found that HBx reduced the activity of COX. This in turn affected mitochondrial respiration, leading to mitochondrial dysfunction [67]. Xie et al., transferred the HBx protein gene into the human liver cell line HL7702 and found that HBx activated the inflammasome NLRP3 in HL7702 cells and promoted pyroptosis via the mitochondrial ROS pathway under OS conditions [68]. Similarly, Gao et al., suggested that HBx regulated MPTP and increased ROS levels in HBx-transgenic HL7702 cells, making the cells become more sensitive to OS, while cyclosporine A (CsA) blocked MPTP and attenuated the OS [69].

Additionally, HBV infection also increased the mitochondrial uptake of Ca^2+^. Subsequently, the excessive accumulation of Ca^2+^ in mitochondria further activated MPTPs and changed the permeability of the mitochondrial membrane [70]. In contrast, blocking the mitochondrial uptake of Ca^2+^ inhibited the elevation of HBx and the replication of HBV [71]. Jabeen et al., constructed HBV-infected HepG2 and HepAD38 cell models and found that Ru360, an inhibitor of mitochondrial calcium uniporter (mCU), inhibited the mitochondrial uptake of Ca^2+^, thereby combating HBV infection [72]. The characterizations of mitochondrial dysfunction in viral hepatitis are summarized in Table 5.

## 5. Problems and Prospects

The integrity of the mitochondrial structure and normal function is necessary for the survival of hepatocytes. The mechanisms of action are extensive and complex in chronic liver disease, but mitochondria are the most vulnerable organelles, and their role in chronic disease is increasingly recognized. Among the various mechanisms of liver disease, mitochondrial dysfunction is a common form and closely related to liver disease, especially for chronic liver disease. Currently, there are many studies on the relationship between mitochondrial dysfunction and chronic liver disease, but an effective and feasible method to detect mitochondrial damage has not yet been established. Therefore, there are still unknown challenges encountered by mitochondria-targeted therapy. More efforts are needed to address the prevention and treatment of chronic liver disease via the mitochondrial pathway.

With the deepening of basic theoretical research on mitochondrial structure and function and the development of biological research methods, people can apply a variety of modern omics technologies to more deeply and thoroughly explore the relationship between mitochondrial dysfunction and chronic liver disease. For example, the development of high-throughput sequencing technologies and advances in computational biology and bioinformatics have led to a rapid increase in multi-genome-wide association studies (GWAS), which have improved our knowledge of genetic biomarkers for liver disease diagnosis and pathogenesis [73]. To date, the National Human Genome Research Institute (NHGRI)-GWAS has reported 24 GWAS studies and more than 100 genetic variants associated with NAFLD traits [74]. This will help to elucidate new pathological mechanisms and provide more feasible ideas for targeting mitochondria in the diagnosis and treatment of chronic liver disease.

## Figures and Tables

**Table 1:** Characterizations of mitochondrial dysfunction in DILI.

Types of Dysfunction	Specific Characterization	References
Morphological structure	Outer membrane is ruptured, membrane permeability is increased, and membrane potential is decreased.	[17,18,19,20,21,22,23,24,25]
Energy metabolism	ATP synthesis is decreased.	[17,18,22,25]
ROS	Oxidative stress occurs, GSH is decreased, and ROS is increased.	[16,17,18,19,20,21,22,23,24,25,27,28]
mtDNA	mtDNA is damaged by nucleases or ROS.	[17,23]

**Table 2:** Characterizations of mitochondrial dysfunction in NAFLD.

Types of Dysfunction	Specific Characterization	References
Morphological structure	Mitochondrial membrane integrity is damaged, membrane potential is decreased, membrane permeability changes, and membrane lipid composition changes.	[30,31,32,36,37]
Energy metabolism	Fatty acid synthesis is increased, fatty acid oxidation is decreased, and fatty acid accumulation occurs.	[29,30,31,32,33,34,35,36,37]
ROS	Oxidative stress occurs, MDA and ROS are increased.	[30,32,35,36,37]
mtDNA	Mitochondrial biosynthesis is attenuated and mtDNA content is reduced.	[35]
Autophagy	Mitophagy is inhibited.	[13]

**Table 3:** Characterizations of mitochondrial dysfunction in AFLD.

Types of Dysfunction	Specific Characterization	References
Morphological structure	Mitochondria are swollen, the inner membrane is destroyed, the cristae have disappeared, and the membrane potential is decreased.	[41,43,45,46]
Energy metabolism	Fatty acids have accumulated and ATP synthesis is decreased.	[39,40,41,42,43,46]
ROS	GSH and SOD are decreased, and ROS is increased.	[39,41,42,44,45,46,47]
mtDNA	mtDNA copy number is reduced.	[41,45]
Autophagy	Mitophagy is inhibited.	[13,41,43,44]

**Table 4:** Characterizations of mitochondrial dysfunction in HCC.

Types of Dysfunction	Specific Characterization	References
Morphological structure	Mitochondrial uncoupling protein inhibits apoptosis of liver cancer cells by reducing mitochondrial membrane permeability.	[53]
Energy metabolism	Fatty acid oxidation is enhanced. Mitochondrial respiratory chain complex I (MRCC-I) and ATP synthase E subunit are increased.	[54,55,56]
ROS	During the development of HCC, ROS is increased.	[50,51]
mtDNA	mtDNA is damaged by ROS, resulting in mitochondrial gene mutation.	[49,50]

**Table 5:** Characterizations of mitochondrial dysfunction in viral hepatitis.

Types of Dysfunction	Specific Characterization	References
Morphological structure	Mitochondria lose their original shape, swell, cristae disappear, membrane permeability changes, and membrane potential is decreased.	[64,69,70,71,72]
Energy metabolism	ATP content is decreased.	[65,67,69]
ROS	ROS is increased and SOD is decreased.	[64,67,69]
mtDNA	mtDNA is damaged by ROS.	[65]

## Data Availability

All data supporting the findings of this study appear in the submitted manuscript or are available from the corresponding author upon reasonable request.
